# Viewpoint on preliminary analysis of artificial intelligence-based thyroid nodule evaluation in a non-subspecialist endocrinology setting in thyroidology

**DOI:** 10.1590/1806-9282.20260576

**Published:** 2026-08-17

**Authors:** Ilker Sengul, Demet Sengul

**Affiliations:** 1 Giresun University, Faculty of Medicine, Thyroidology Unit – Giresun, Turkey.; 2 Giresun University, Faculty of Medicine, Division of Endocrine Surgery – Giresun, Turkey.; 3 Giresun University, Faculty of Medicine, Department of General Surgery – Giresun, Turkey.; 4 Giresun University, Faculty of Medicine, Department of Pathology – Giresun, Turkey.

The recent study regarding the implementation of artificial intelligence-based decision support systems (AI-DSS) in non-subspecialist endocrinology settings provides a timely and essential contribution to the evolving landscape of digital for thyroidologists^1^. By shifting the focus from high-expertise tertiary centers to the point-of-care ultrasound (POCUS), environment of general endocrinology, the authors highlight a critical translational gap: the performance of deep learning algorithms in low-prevalence real-world populations. As such, while the study's conclusion that non-adjunct artificial intelligence (AI) use may lead to risk overestimation is sobering, several methodological and conceptual nuances warrant further discourse. A primary concern lies in the retrospective nature of the AI analysis conducted on static images captured by non-subspecialists. Furthermore, ultrasound is inherently a dynamic, operator-dependent modality; the "best" diagnostic frame is often selected based on the clinician's subjective impression. If the initial image acquisition by a general endocrinologist lacked the most suspicious features of a nodule, even the most sophisticated AI-DSS would be fundamentally limited by the input data. Moreover, the study notes that the AI system detected microcalcifications significantly more frequently than clinicians (10.7 vs. 1.3%), yet it missed comet-tail artifacts—a hallmark of benignity—in almost all cases where clinicians identified them, which suggests that the algorithm may be overly sensitized to high-frequency echoes without the contextual "gestalt" required to differentiate malignant microcalcifications from benign artifacts. Additionally, the observed low agreement (Kappa=0.026) between the general endocrinologists and the AI-DSS regarding the American College of Radiology Thyroid Imaging Reporting and Data System, American College of Radiology Thyroid Image Reporting and Data System, categorization raises questions about the "black-box" nature of these tools when deployed outside their training distributions. The authors correctly identify that AI models trained in high-malignancy settings tend toward a conservative, high-sensitivity bias. However, without a definitive gold standard (histopathology) for the majority of the non-referred cohort, it remains speculative whether the AI was "overestimating" risk or if the generalists were potentially "under-stratifying" subtle features. Of note, the reliance on Category II results for only a small subset of referred nodules limits the ability to accurately evaluate the AI's diagnostic accuracy. While the authors advocate for the adjunctive use of AI, the risk of automation bias—where clinicians may eventually defer to the AI's "high suspicion" labels—could paradoxically increase the burden on subspecialized thyroid nodule clinics rather than alleviating it. Future research must focus on "human-in-the-loop" prospective designs in which the AI's influence on the clinician's real-time decision-making is measured rather than on post hoc comparisons of independent assessments. In essence, the authors have eloquently demonstrated that AI is not a "plug-and-play" solution for every clinical tier. For AI to become a true asset in thyroidology^2-5^, and general endocrinology, algorithms must be recalibrated for screening-level prevalence, emphasizing high negative predictive values to safely reduce unnecessary referrals. This issue merits further investigation. We thank Fernández Velasco et al.^1^ for their valuable study on the pivotal role of AI in thyroid nodule evaluation for thyroidologists.

## Data Availability

The datasets generated and/or analyzed during the current study are available from the corresponding author upon reasonable request.
